# Exploring the links between sonochemistry and sonomechanobiology

**DOI:** 10.1016/j.ultsonch.2025.107697

**Published:** 2025-12-03

**Authors:** Timothy J. Mason, Mircea Vinatoru

**Affiliations:** a School of Health, Coventry University, Coventry CV1 5FB, UK; b Faculty of Chemical Engineering and Biotechnologies, National University of Science and Technology, Politehnica Bucharest 011061 Bucharest, Romania

**Keywords:** Bioreactions, Cavitation, Cell biology, Standing waves, Ultrasound

## Abstract

Sonomechanobiology concerns the ways in which vibrational energy can stimulate biological cells. It is a multi-disciplinary subject involving contributions from fields including chemistry, food science, microbiology, medicine and physics. Early studies of the effects of ultrasound on living tissue can be traced back to the 1920′s but in those days, without the aid of modern specialised equipment, detailed explanations were not possible. The more recent work on the stimulation of cells has been of particular interest to microbiologists and medical physicists while sonochemists have not really been involved. But sonochemistry has an important part to play in the developing subject of sonomechanobiology and this opinion paper will re-examine those early studies which can be considered to be precursors to both sonomechanobiology and sonochemistry.

## 1 Introduction

Sonomechanobiology is a relatively new term which was defined by Ambatto and Yeo in 2023 as “A bringing together of the many and various ways in which vibrational energy can stimulate cells” [1]. In March 2025 a workshop was held at the Lorentz Center in Leiden University entitled “Sonomechanobiology: Understanding High Frequency Mechanostimulation” [2] which brought together experts from different scientific backgrounds that included microbiology, chemistry, medicine and physics. From this meeting it became clear that in order to gain a better understanding of the effects of ultrasound on living tissue it was necessary that these disciplines should collaborate. While everyone was keen to develop links there were some difficulties due to the specialised nature of each discipline and the subject specific terminologies associated with each. In very broad terms the subject areas involved sonomechanobiology are:

• Techniques for applying ultrasound to living material
• Methods of quantifying the resulting biological effects
• The interpretation of such effects
• Exploring applications for any observed cell stimulation

Generally, scientists and medics will specialise in one or two of these fields although a few will already be working in several of them. However, the biologists, chemists, biomedical engineers, medics and physicists who have interests in sonomechanobiology do not tend to meet up very much, they will attend different sorts of conference and also read different journals. In the opinion of the authors, it will be necessary to bring these disciplines together in order to further a greater understanding of sonomechanobiology.

An important question is what can sonochemists bring to the party? In this opinion paper we will review some early investigations into the effects of ultrasound on biological materials which could, in retrospect, be seen as the precursors to sonomechanobiology. The earliest date from the 1920′s when the scientific instrumentation was not as well developed as that which is available today. Those early observations and others which followed, provide subjects for re-investigation by multidisciplinary groups and this will help towards a greater understanding of sonomechanobiology.

## 2 Sonochemistry and biology

### 2.1 Early history of the study of the biological effects of ultrasound 1925–1960

The first paper on the effects of ultrasonic irradiation on biological systems was published in 1927 by Robert Williams Wood and Alfred Lee Loomis entitled “The physical and biological effects of high frequency sound waves of great intensity” [3]. Robert Wood had first presented this research work in October 1926 at a meeting to commemorate the fiftieth anniversary of the founding of Johns Hopkins University. The published paper was subtitled “Communication No 1 from the Alfred Lee Loomis Laboratory, Tuxedo, N.Y.” indicating that the work was not carried out at a university but in an independent privately owned laboratory.

The background to the formation of this laboratory is an important milestone in the history of sonochemistry. Robert Wood was a professor of physics at Johns Hopkins University and Alfred Loomis was a New York finance banker and multimillionaire who had a passionate interest in the sciences. Wood was 20 years older than Loomis and they had met during the First World War when they were both serving in the army as scientists at the Aberdeen Proving Ground (APG) in Maryland. After the war Loomis had used some of his considerable wealth to convert a large garage at his house in the small town of Tuxedo Park, in Orange County, New York into a laboratory [4]. The families of both Loomis and Wood both spent summer holidays in Orange County and it was here, in the 1920′s that they met again and began scientific research together. In order to pursue work involving ultrasound Loomis purchased two generators from the laboratories of General Electric in Schenectady New York. These were used to drive quartz transducers with an ultrasonic output of 2 kW over a frequency range of 100 to 700 kHz. This apparatus was built at Schenectady and then transported and installed in the laboratory in Tuxedo Park. Later, as the studies expanded, more space was needed and Loomis purchased a nearby large stone-built mansion (Tower House, also in Tuxedo Park) and had it converted into a research facility which was used from 1926 until 1940 and became known as the Loomis Laboratory.

Towards the end of the Wood and Loomis paper [3] the effects of high frequency vibrations on living matter were summarised as follows:

• Filaments of living algae spirogyra suspended in water were torn to pieces and the cells ruptured.
• Blood cells in physiological saline were rapidly destroyed by exposure to these waves and the turbid liquid became clear and red.
• Small fish and frogs were killed in one or two minutes.
• Mice were more resistant to the effects and, although barely able to move after 20 min exposure, they recovered fairly rapidly.

The report of the work concluded: “*It would seem that interesting and important avenues of approach have been opened by this, the first communication from the Alfred Lee Loomis Laboratory, Tuxedo, NY*.”.

Alfred Loomis continued his investigations into the biological effects of ultrasound with a zoologist, Edmund Newton Harvey, from the Physiological Laboratory at Princeton University and in 1928 published a paper in Nature [5]. They reported some work carried out with the original high-power Wood and Loomis equipment on aqueous suspensions of two small motile microorganisms *Euglena* and *Paramecium*. The former uses a flagellum for movement and the latter cilia. Each was enclosed in a capillary tube sealed at both ends and when one end was subjected to intense vibrations the organisms were driven into bands regularly spaced at about 2 mm along the tube, from which they are unable to escape by swimming. These bands are the nodes of stationary waves set up in the capillary. Within these nodes the organisms were unharmed but if the experiment was carried out in a test tube where convection currents forced them outside of the protective nodes, they were killed. A similar experiment found that red blood cells were also carried to the nodes created in a capillary tube. The paper made reference to the previous observation of Wood and Loomis that red blood cells were destroyed when subjected to ultrasound in suspension in a test tube because no nodes were formed [3]. The term *“laking”* was used in these and other papers to describe the destruction of the red cells to form a homogeneous solution. In a test tube the destruction occurred in one minute, during which the average temperature of the fluid did not reach 37 °C. It was therefore clear that the destruction was not caused by any change in the bulk temperature induced by ultrasound but rather by some form of mechanical effects.

During this period (in 1928) Schmitt, Olson and Johnson, who were based at the chemistry department of the University of California at Berkeley, were pursuing similar studies. They published preliminary work entitled “Effects of high frequency sound waves on protoplasm” [6]. It is interesting to note that, at the end of this paper, they mentioned that the Nature paper of Harvey and Loomis had been published after they had submitted their manuscript, to make it clear that their own work was an independent study. The Californian group used quartz transducers operating at 750,000 cycles per second to transfer vibrations into a crystallising dish containing xylene. This acted as an ultrasonic bath and any test tube dipped into the xylene would transfer energy to its contents. Using this technique free-swimming Triturus larvae (newt tadpoles) suspended in a test tube were rapidly killed as were worm-like Spirostoma. These findings were in agreement with those of Wood and Loomis.

Further work on the biological effects of ultrasound was published in the same year by Harvey and Loomis together with Harvey's wife Ethel Browne Harvey [7]. She worked in the same Princeton laboratory as her husband and also at the Marine Biological Laboratory at Woods Hole in Massachusetts. In this study the biological effects were observed under a microscope, but the high-power ultrasonic equipment used in the earlier Wood and Loomis work was not suitable for this. They constructed an apparatus consisting of a 75-Watt high frequency oscillator driving a quartz crystal at 40,000 cycles per second. The biological material was mounted directly on the crystal which acted as a microscope slide.

Using this apparatus, they studied the effects of ultrasonic waves on a leaf of the waterweed Elodea, an oxygenating freshwater plant often used in aquariums. Observations revealed that only certain areas in the leaf show the characteristic whirling of the chloroplasts described in their previous paper [5]. The whirling areas did not correspond to any particular region of the quartz surface because, when the leaf was moved to a new position on the oscillating crystal, it did not change the areas where whirling occurred. In a separate experiment, when the position of the specimen on the crystal was not changed, any slight alteration in frequency caused the patterns to shift. Chloroplasts in the cell could be made to rotate slowly or rapidly, clockwise, or counterclockwise, in one vortex or in a series of vortices. By increasing the intensity, the leaf of Elodea could be agitated so violently that the chloroplasts themselves were broken into a fine green emulsion which completely filled the cell. It was established that this was not the result of any heating generated by the quartz resonator by placing crystals of ethyl stearate on the Elodea leaf during an experiment. These crystals have a melting point of 30–31 °C and did not melt even after 15 min of sonication.

Various other observations were made including the effects of ultrasound on fertilized fish eggs. When eggs of a small killfish (genus fundulus) are mounted on the crystal they can be very violently agitated to the extent that the oil drops and granules within them are “made to dance”. The yolk was thoroughly stirred and the surface of the protoplasm was seen to move and bend. If these treated eggs were allowed to proceed to hatch the fish development appeared to be normal. However, if acoustic power was amplified beyond a certain level the increase in agitation caused the eggs to rupture.

**Fig. 1:**
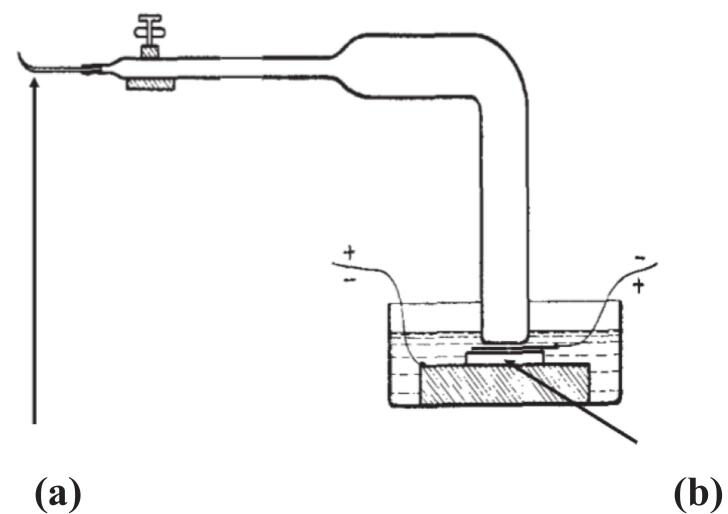
Apparatus for introducing ultrasound through a vibrating tip (adapted from [8]). (a) strip of glass, drawn to a thread at end. (b) quartz transducers transfer vibrations to glass strip via a sonicated crystallising dish containing xylene.

In 1929 Francis Schmitt, one of the co-authors of a previous paper from the University of California [6], published a paper entitled “Ultrasonic micromanipulation” in which he described a different approach to the way ultrasonic vibrations could be introduced directly to a cellular species [8]. A flat strip of glass, drawn to a thread at one end, was used, to transfer vibrations from the ultrasonic bath described previously [6] through a glass strip to a finely drawn out tip (Fig. 1). Planarian worms lightly touched by the tip were instantly burned. By modification of the tip, it became possible to insert the point into an amoeba and to observe the effects on its protoplasm. Preliminary experiments indicated that insertion of the needle produced an alteration of the viscosity of the protoplasm. Provided that the treatment was not too intense or too prolonged, the effects appeared to be reversible. However more intense treatment produced local injury, and in extreme cases complete internal disorganization, or even disruption, of the cell.

In the same year (1929) C. H. Johnson from the University of California, reported the lethal effects of ultrasonic radiation on protozoa and red blood cells [9]. Separate aqueous samples of the protozoa Stentor, Spirostomum, Blepharisma and Paramecium were irradiated using the same quartz-crystal oscillator described previously [6]. Vigorous cavitation of air bubbles was observed under atmospheric pressure and in each case the protozoa were destroyed in 30 s. Experiments were performed under applied pressures varying from 10 to 60 lb. per sq. inch (69–414 kPa) using an oxygen tank connected to the test tube. The pressure did not interfere with sound propagation through the liquid; but the appearance of bubbles of visible size was entirely prevented. At a critical pressure of 60 lb. per sq. inch (414 kPa), when bubble generation stopped, protozoa were almost unaffected by 2 min of radiation. It was noted that the same overpressure also prevented the ultrasonic oxidation of aqueous iodide to iodine [10]. Using these observations Johnson drew attention to the great importance of the formation of minute bubbles of gas within the liquid as the source of activity induced by ultrasonic radiation.

Similar conclusions were drawn from studies of the effects of ultrasound on the red blood corpuscles of a rabbit suspended in physiological saline at different pressures [9]. As with protozoa, when the critical pressure was reached the corpuscles collected at the nodes in “pretty patterns” and were not destroyed even after an exposure of several minutes. In the paper there is a mention of a visitor to Berkeley while this work was in progress. He was F. J. W. Roughton of Cambridge University UK, who had research interests in blood physiology. Roughton suggested some new ultrasonic experiments which involved reducing the overpressure on suspensions of blood corpuscles in saline well below atmospheric in order to remove oxygen, carbon dioxide and dissolved air. The results of exposure of such suspension to ultrasonic radiation, under vacuum, produced “sound patterns” from the standing waves generated within the test tube and led to sedimentation after about 5 min. It seemed that there was no appreciable amount of cell destruction because when the test-tube was returned to atmospheric pressure and shaken for a minute or two with air to saturate the corpuscles with oxygen the red colour returned very quickly. However, when the aerated mixture, at atmospheric pressure, was again subjected to ultrasound all the cells were destroyed.

Johnson suggested that the intense mechanical agitation produced by ultrasound at the liquid cell interfaces was primarily responsible for the destructive effects. Further that the production of a powerful oxidizing agent in water containing dissolved oxygen would accelerate the killing of micro-organisms but this was thought to be a secondary effect. His observations on the behaviour of red blood cells were interesting in that it posed the question as to whether the oxygen and carbon dioxide carried inside the cells could take any part in their destruction. The paper concluded with the statement that “*It seems likely that the lethal action is from without rather than from within; external rather than internal*”.

In 1928 Harvey and Loomis reported that the light emitted by luminous bacteria was dimmed when exposed to high power ultrasonic waves [5]. Harvey had a long-time interest in the chemistry of bioluminescence and, some years earlier, had published a paper on the bioluminescence of the firefly [11]. It was therefore a natural progression for him to continue this by working with luminous bacteria. In 1929, together with Loomis, he reported that sonication at 375 kHz and 19℃ caused a reduction in light emission from a seawater suspension of rod-shaped *Bacillus Fisheri* [12]. This could be related directly to the killing of these bacteria. Two important points regarding the experiment were emphasised in this paper:

• Great care was needed to maintain the temperature of the bacteria suspension in sea water at 19℃ or below because higher temperatures would kill them even in the absence of ultrasound.
• The vessel in which the sample was suspended should be a round bottomed tube and constant agitation was required so that no standing waves could be set up. In the presence of standing waves, the bacteria could be drawn into nodes, where they would remain unharmed, as happens in capillary tubes.

The final sentence of the paper predicted a poor future for the commercial exploitation of sonication (which they referred to as “raying”) and this read: “*In conclusion we can state that, under proper conditions of raying, luminous bacteria can be broken up and killed by sound waves of approximately 400,000 cycles per second and the solutions sterilized, but the method is not one of any practical or commercial importance because of the expense of the process*”. At the time that this was written the conclusion was probably correct since ultrasound generators were large pieces of equipment which had originally been used for underwater echo-sounding and were not readily accessible to the general scientific community. However Harvey and Loomis were not to know that as ultrasonic technology developed so laboratory equipment would become smaller, more accessible and less expensive. Therefore their gloomy prognosis proved to be incorrect.

Harvey and Loomis continued their exploration of the effects of ultrasound on living cells with the use of a newly developed high speed camera capable of taking 1200 pictures a second through a microscope objective. [13]. The cells chosen for study under sonication were the unfertilized eggs of sea urchin (diameter 75 µm). Photographs indicated that the disintegration of the eggs occurred in less than 1/1200 s. They were drawn out into spindle or tadpole shapes which suggested that rapid movements of the fluid were tearing the eggs apart. They suggested that the destruction of cells by ultrasound might be the result of the effects of tiny cavitated gas bubbles outside the cell. No cavitated air bubbles were shown in the photographs and so they concluded that the cavitation events themselves must be sub-microscopic.

A general survey of the effect of ultrasonic waves on biological material was published by Harvey in 1930 [14]. He was the sole author, but his affiliations were listed as the Loomis Laboratory at Tuxedo Park and the Physiological Laboratory at Princeton, however he noted that almost all of the experiments were performed at the Loomis laboratory. He suggested that the effects of ultrasonic waves on biological material could be grouped into five categories:

• Whirling of the protoplasm.
• Displacement of small particles.
• Cytolysis (disruption) of whole cells.
• Disintegration of small bodies like chloroplasts within the cells.
• Stimulation of cells.

Local heating was discounted as a source of these effects because crystals of ethyl stearate did not melt under these conditions (see [7]). It was concluded that these effects must have been caused by the physical and chemical effects of ultrasound.

The break-down of biological material resulting from exposure to ultrasound also offers a method for extracting cell contents and in 1946 this technique was used to explore the extraction of bacterial enzymes [15]. It was a particularly useful technique when limited quantities of bacteria were available. In addition, since irradiation of bacteria by ultrasound can be carried out under completely aseptic conditions, it was possible to analyse pathogenic bacteria. At the time the method was considered to have two drawbacks: (1) the construction of the apparatus was expensive, and (2) not all bacteria can be disintegrated by ultrasound. Later developments in technology resulted in the production of ultrasonic cell disruptors which have now become standard pieces of apparatus in microbiology laboratories [16].

## 3 The development of standing wave technology from the 1930′s

**Fig. 2:**
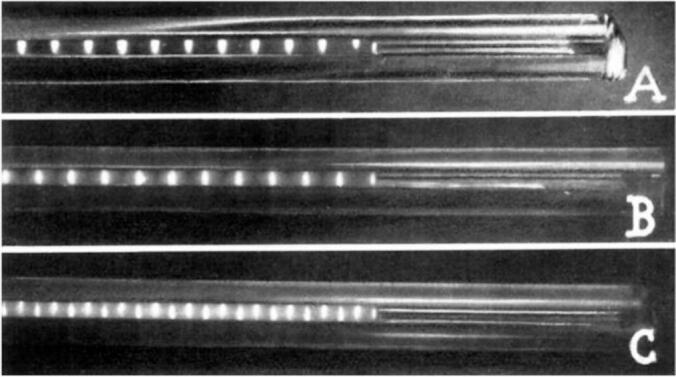
Separation of particles in a standing wave (adapted from [17]). (A) quartz particles (density 2.66) in water collect at pressure antinodes. (B) toluene (density 0.87) in water moved to pressure nodes. (C) separations also occurred in a mixed system with alternate quartz and toluene collected at the antinodes and nodes with separation between each at ¼ wavelengths.

Standing waves had been identified as an important component of the effects of ultrasound on biological material. These were also of great interest to physical chemists working on the coagulation and separation of materials. In 1936 Sollner and Bondy from the Chemistry Department of University College London showed how materials in a liquid could be aggregated and separated in a horizontal capillary tube [17]. The separations were caused by stationary waves in the liquid a phenomenon which the authors noted was the same as Kundt’s dust figures produced by ultrasound in cylindrical liquid columns [18]. A glass capillary with a slight U-shape in its centre was filled with the mixture to be separated. Ultrasound was generated by a piezoelectric plate at the base of an oil bath which produced a “fountain” at the surface. If the U-shaped part of the tube is dipped into the oil fountain (with the two ends of the tube extended horizontally sideways) stationary waves are produced along it. Any suspended material in the liquid in the tube collects in the standing waves at half wavelengths along it. The positions of the separated material at a node or antinode depended upon the type of material used (Fig. 2). It was shown that quartz particles collected at the antinodes whereas toluene droplets moved to the antinodes. A striking demonstration of this type of separation was revealed when a mixture of quartz and toluene were exposed to a standing wave and the two materials collected in alternating individual zones in the tube at ¼ wavelengths distances.

In 1952 Goldman and Lepeschkin from the Naval Medical Research Institute, Bethesda, Maryland published a report on the effects of standing sound waves on the filamentous green algae spirogyra and other species [19]. The study was carried out using frequencies of 400, 700 and 1,000 kHz and was intended to determine the mechanism by which cavitation damage occurred on living material in suspension. When spirogyra filaments were exposed to a cavitating field in water with a standing wave present, injury to the filaments was widespread and rapid. However, when the spirogyra was trapped in agar gel (the gel does not support cavitation) and placed in a standing wave field there was still damage, but it was much lower and primarily at the nodes of the standing wave. The authors studied several different types of living cell under these conditions and concluded that:

• Exposure of living cells at different positions in a standing wave sound field shows marked positional effects.
• In the presence of cavitation, injury is widespread and rapid.
• In the absence of cavitation, injury occurs slowly at the pressure nodes.
• Mechanical injury to cells can occur in several ways but there is not yet enough information available to characterise these differences.

The authors made the point that cavitation was “*not yet a well understood phenomenon*”. In previous studies by Harvey and Loomis there had been no evidence of any cavitation inside the cell itself [13], [14] probably because the high viscosity of protoplasm. For this reason, it had been concluded that any mechanical injury was produced mainly by cavitation bubbles in the surrounding fluid [20].

### 3.1 Developments in beneficial uses of ultrasound on living systems from 1960

By 1951 interest in the effects of ultrasound on living material had grown so rapidly that there was a need for an extensive data resource on the subject. This was produced in the form of a bibliography which contained 580 references on biological, biochemical and biophysical applications of sonic and ultrasonic vibrations [21]. Two years later Pierre Grabar, from the Pasteur Institute, Paris, reviewed the effects and the mechanisms of action of ultrasonic waves on living matter and cited 343 references [22].

Together with continued interest in the harmful effects of ultrasound on living cells there was also a growing awareness of possible beneficial effects on cells referred to in 1930 by Harvey as the stimulation of cells [14]. The most widely used applications of such stimulation are to be found in therapeutic ultrasound which continues to be the subject of major developments in surgery, drug delivery and wound healing. The amazing results obtained are somewhat beyond the scope of this paper but can be readily accessed through reviews in the medical literature [23], [24], [25], [26], [27], [28]. Cell stimulation has also attracted interest in other fields, which include improvements in bioreactors and some eye-catching possibilities which were, up to a few decades ago, simply research ideas. These are now emerging as important commercial targets all of which have a link with sonomechanobiology.

### 3.2 Ultrasound and agriculture

There is an urgent need to increase food production to meet the problems posed by an expanding world population. One way of achieving this is to improve crop yields by shortening the germination time of seeds and/or increasing the percentage germination. Ultrasonic treatment of seeds offers a “green” method for achieving these objectives.

One effect of the sonication of seeds or sprouts in an aqueous medium is surface cleaning which will remove microbial contamination and so reduce seed decay which is a major problem in agricultural production. Ultrasonic surface cleaning has been explored in the cases of fruits and vegetables and has been applied, more recently to the decontamination of seeds [29].

There are however other effects, in addition to surface cleaning, which can improve germination and, in some cases, boost subsequent growth. This was first reported in the years around 1940 by researchers in Russia who observed the influence of ultrasound on seed germination and plant development [30]. That work is somewhat inaccessible and, probably for that reason, it attracted little attention at the time. However, ten years later in 1950 Victor Tomberg, from the University of Bruxelles published a paper (in French) which described the influence of ultrasound on the germination and root growth of watercress [31]. He employed a frequency of 800 kHz at 0.4 and 2.0 W/cm^2^ to sonicate the seeds, in water, for a period of 10 min. This low dose had a positive effect (83 % germination compared with about 70 % in a control sample) although higher doses were found to have an inhibiting effect with only 36 % germination. By comparing iodine liberation, used as a measure of cavitation, with the root growth of watercress seedlings a relationship was found between the chemical and physiological action of ultrasound. From this relationship it was concluded that the action of ultrasound on the seedlings was the direct result of the effects of cavitation.

One of the factors which can contribute to the breaking of the dormancy of seeds is the uptake of water. Sonication of seeds in water leads to cavitation bubble collapse near the seed surface which can result in the creation of micro-pores and micro-cracks in the protective shell. This will make the seeds more permeable to water and oxygen entry which is also enhanced by cavitation. This was demonstrated by Busnel and Obolensky in 1954 who monitored the penetration of colored water into barley seeds and found an improvement of between 10 and 35 % [32]. Obolensky continued his interests in barley and some years later in 1960 the results of treatment at several different frequencies was published [33]. These confirmed that the increased germination and growth acceleration of barley seeds appeared to be due, at least in part, to an irreversible modification of the permeability of the seed coats. However, if ultrasound was applied for too long it produced traumatic destruction of the cellular structure.

In addition to specific research on seeds there have also been a number of reviews on the effects of ultrasound on plants, one of the earliest of these was in 1963 by Andrew Gordon from the Department of Agricultural Botany, University of Aberdeen in Canada [34]. Nearly 10 years later the same author, who was then working for the UK Forestry Commission, published another review [35]. It is interesting to note that while he proposed that there were certainly significant ultrasonic effects on plants, the causes of them and the inconsistencies in their effects were barely understood at that time.

In 1983 a wider range of the more general botanical effects of ultrasound were reviewed by D. L. Miller which contained over 100 references [36]. Much of the article concerned potential damage to biological material and, as a physicist, he was concerned with the link between these and the measurement of acoustic energy input. The conclusion included the following sentences which indicated the need for further research in the field: *“It is proper to ask what use can be made of all this information on botanical effects of ultrasound and to identify deficiencies in our knowledge. We now have a catalogue of effects, data on the exposure conditions required for them and even a partial understanding of the processes which damage plant tissues exposed to ultrasound. The operative mechanisms and the biophysics of their action on cells have only been qualitatively elucidated, and quantitative observations are needed for a fuller understanding of the processes which damage plant tissue exposed to ultrasound”.*

**Fig. 3:**
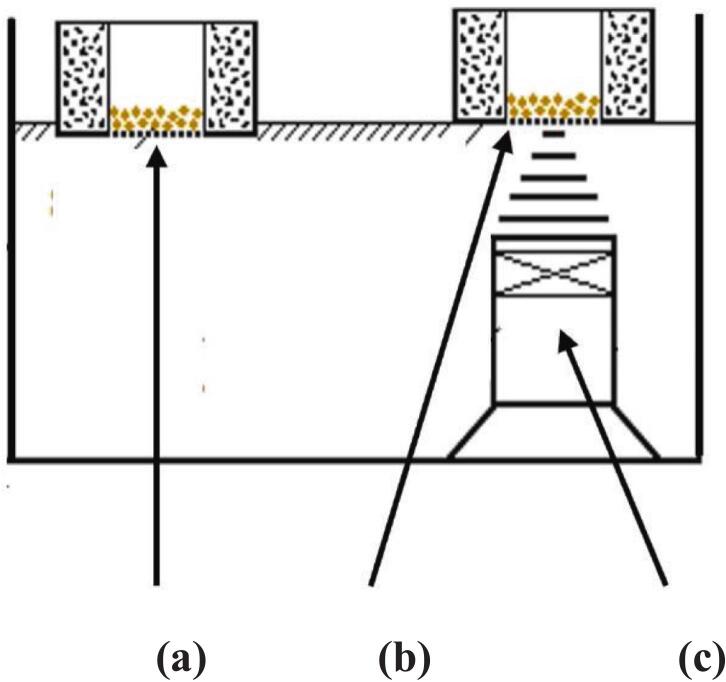
Ultrasound exposure system for radish seeds (adapted from [37]). (a) floating basket (control). (b) floating basket (sonicated). (c) ultrasonic transducer.

Interest in seed treatments continued after the publication of these reviews and in 1990 Shimomura reported the effect of ultrasonic irradiation on radish seeds after germination to monitor any differences in subsequent growth [37]. His approach involved two seedbeds floating on the surface of a thermostatted water bath (22 L) submerged in which was a piezoelectric ceramic transducer (30 mm diameter at a frequency of 700 kHz) (Fig. 3).

The ultrasound was directed at one seedbed and the other seedbed was outside of the influence of ultrasound and acted as the control. At the same temperature the lengths of ultrasonically irradiated root were 13 to 16 % greater than the control. The results showed that the weight increase of seeds due to absorption of water and the growth of roots were both accelerated by the effects of ultrasonic irradiation and were not related to thermal action.

A review on seed germination and seedling development have been published from the University of Minho, Portugal, entitled “Seed germination and seedling development assisted by ultrasound” which contains 116 references [38]. It was shown that ultrasound stimulated both the percentage germination and subsequent vigour of seedling development for a variety of species. It was also reported that this treatment was effective in improving the germination percentage of older seeds. The authors suggested that further investigations were needed at a molecular level to elucidate the mechanisms by which ultrasound acted upon seeds. This was the subject of a second paper from the same group, relating to the mechanism of improvements in seed hydration induced by ultrasound [39]. Hydration is a key process in germination and two factors were identified as contributors to this improvement:

• cavitation damage to the microstructure of the seed coatings making them more porous (revealed by SEM) and
• changes to water transport between cells through alterations to channel proteins that form pores in the membrane of biological cells

### 3.3 Ultrasound and fish farming

A remarkable influence of ultrasound on fish egg hatching was reported in 1992 [40]. When loach *(Paramisgurnus dabryanus)* eggs were exposed to ultrasound (1 MHz) for 35 min, three times a day there was a reduction in hatch time from 72 to 60 h. This alone is of industrial importance for fish-farming but there were two additional benefits. Treatment with ultrasound increased the percentage of the eggs which hatched and also, once hatched, the fish had a higher survival rate.

Another approach to increasing the productivity of fish farms is to improve fish digestive efficiency so that they gain weight more rapidly. Ultrasound has been used to enhance the transport of arginine or glutamine across the embryo membranes of zebrafish *(Danio rerio)* through the process of sonophoresis [41]. These amino acids are known to stimulate the development and maturation of the fish gut so that the treated embryos exhibited higher growth rates and there is also an improved growth of the fish larvae.

### 3.4 Ultrasound and bioreactions

#### 3.4.1 Reactions induced by bacteria

**Fig. 4:**
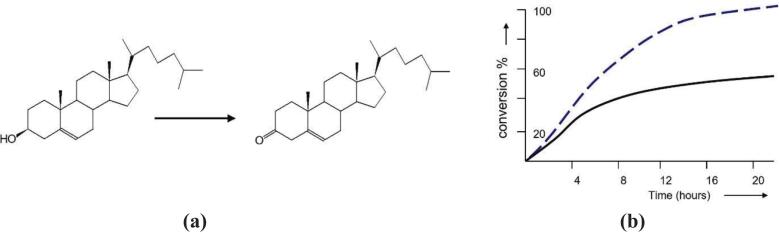
Ultrasonically assisted oxidation of cholesterol with Rhodococcus erythropolis.(adapted from [42]). (a) Scheme for cholesterol oxidation using Rhodococcus erythropolis. (b) Conversion % without ultrasound (^________^) and with ultrasound (− − − −).

In 1988 Bar published a report on enhanced microbial activity induced by ultrasound relating to the oxidation of cholesterol (Fig. 4) [42], with the gram positive bacterium *Rhodococcus erythropolis*. Using cholesterol at a concentration of 2.5 g/l, at 26℃ a 40 % increase in yield was obtained with ultrasound at 20 kHz (2.2Wcm^−2^ pulsed at 5 sec every minute). The cell structure was preserved throughout the experiment. In a subsequent paper from the same author the dehydrogenation of hydrocortisone by gram positive *Arthrobacter simplex* ATCC 6946 was investigated at 20 kHz [43]. Sonicated reactions under stirring were compared with stirring alone and only a small enhancement was observed using freely suspended cells. However, the results were improved when the bacteria cells were immobilized on calcium alginate beads. It was suggested that this enhancement was associated with ultrasound–facilitated diffusion of the substrates (oxygen and hydrocortisone) within the gel beads.

In 1992 Sinisterra published an overview of applications of ultrasound to biotechnology which, at the time, was a relatively new field of research [44]. He pointed out that low intensity ultrasonic waves could modify cellular metabolism or improve the turnover number of enzymes. These improvements were the result of increased mass transfer of reagents and products through a boundary layer to the bacteria or further into the interior of the cell through the cellular wall and membrane. Although it contained only 31 references, it provided a baseline for the future development of bioreactions and bioreactors.

#### 3.4.2 Bioreactors

One of the first positive results for use of ultrasound to promote a bioreaction on a large scale was the acceleration of the anaerobic digestion of sewage sludge [45]. The degradation process begins with bacterial hydrolysis of the input material. This process is hindered by the presence of any agglomerates because these will shield biodegradable content. However, pre-treatment of the feedstock with ultrasound at a frequency of 31 kHz and at high acoustic intensities will break down any agglomerates resulting in an accelerated hydrolysis rate as evidenced by an increase of Chemical Oxygen Demand in the sludge supernatant.

In the context of sonomechanobiology however the main interest is in the acceleration of the bioreaction itself rather than purely mechanical effects and this normally involves low intensity rather than high powered ultrasound. In 2000 Schläfer et al reported some improvements in the biological activity within a bioreactor produced by low energy ultrasound [46]. A reactor was built to represent fermentation of the process-water from wine or juice production. A synthetic glucose medium was used to simulate the wastewater and the yeast *Saccharomyces cere*v*isiae* C-2324 was employed. Ethanol production without ultrasonic treatment varied between 3 and 12 g/l. At an ultrasound frequency of 25 kHz and a power input of intensity 0.3 W/l, the ethanol concentration increased to values from 18 to above 30 g/l.

In 2003 Christi presented a more general review of the uses of ultrasound for enhanced microbial productivity containing 50 references in which he used the term “Sonobioreactor” [47]. 6 years after this Gogate et al published a review with 100 references of the types of reactors available for larger scale biochemical engineering/biotechnology [48].

## 4 Future prospects for sonochemistry links with sonomechanobiology

A definition of sonomechanobiology used at the start of this opinion paper was that it was a bringing together of the many and various ways in which vibrational energy can stimulate cells [1]. In 2017 a review made the point that interactions between ultrasound and microorganisms, particularly at sub-lethal levels to stimulate activity, are complex and not well understood [49]. That paper was directed towards fermentation reactions which is where many academic and industrial groups continue to engage in active research.

There is clearly a need for more conversation between the different interest groups involved in sonomechanobiology. The most obvious way to achieve this is to organise interdisciplinary meetings where the proportion of each group attending is similar. The current situation parallels in many ways what happened in the early meetings of the European Society of Sonochemistry. From the very first meeting in 1990 the conferences were (and still are) deliberately organised as unified sessions with no parallel sessions. In this way the different areas of applied ultrasound are made available to all delegates. It was difficult at times to understand all of the presentations, but some form of collaboration was achieved.

A possible way forward to achieve collaboration in sonomechanobiology might be that we revisit some of the studies involving the effects of ultrasound on biological material outlined above using modern technologies. This will not only cement the links between sonochemistry and other disciplines, but it might also lead on to new avenues of research in therapeutic ultrasound. A possible way to engage broader industrial interest would be to arrange for some targeted – but not overly technical − presentations to agriculture and medicinal themed conferences.

We should all welcome the development of an interdisciplinary approach to sonomechanobiology as an important step towards understanding the mechanisms underlying life itself..

## CRediT authorship contribution statement

**Timothy J. Mason:** . **Mircea Vinatoru:** Writing – review & editing.

## Declaration of competing interest

The authors declare that they have no known competing financial interests or personal relationships that could have appeared to influence the work reported in this paper.
